# Kinetoplastid Specific RNA-Protein Interactions in *Trypanosoma cruzi* Ribosome Biogenesis

**DOI:** 10.1371/journal.pone.0131323

**Published:** 2015-06-29

**Authors:** Khan Umaer, Noreen Williams

**Affiliations:** Department of Microbiology and Immunology & Witebsky Center for Microbial Pathogenesis and Immunology, University at Buffalo, Buffalo, New York, United States of America; Instituto Butantan, Laboratório Especial de Toxinologia Aplicada, BRAZIL

## Abstract

RNA binding proteins (RBP) play essential roles in the highly conserved and coordinated process of ribosome biogenesis. Our laboratory has previously characterized two essential and abundant RBPs, P34 and P37, in *Trypanosoma brucei* which are required for several critical steps in ribosome biogenesis. The genes for these proteins have only been identified in kinetoplastid organisms but not in the host genome. We have identified a homolog of the TbP34 and TbP37 in a *T*. *cruzi* strain (termed TcP37/NRBD). Although the N-terminal APK-rich domain and RNA recognition motifs are conserved, the C-terminal region which contains putative nuclear and nucleolar localization signals in TbP34 and TbP37 is almost entirely missing from TcP37/NRBD. We have shown that TcP37/NRBD is expressed in *T*. *cruzi* epimastigotes at the level of mature mRNA and protein. Despite the loss of the C-terminal domain, TcP37/NRBD is present in the nucleus, including the nucleolus, and the cytoplasm. TcP37/NRBD interacts directly with Tc 5S rRNA, but does not associate with polyadenylated RNA. TcP37/NRBD also associates *in vivo* and *in vitro* with large ribosomal protein TcL5 and, unlike the case of *T*. *brucei*, this association is strongly enhanced by the presence of 5S rRNA, suggesting that the loss of the C-terminal domain of TcP37/NRBD may alter the interactions within the complex. These results indicate that the unique preribosomal complex comprised of L5, 5S rRNA, and the trypanosome-specific TcP37/NRBD or TbP34 and TbP37 is functionally conserved in trypanosomes despite the differences in the C-termini of the trypanosome-specific protein components.

## Introduction

The early branching eukaryotic pathogens *Trypanosoma cruzi*, *Trypanosoma brucei* and *Leishmania* spp (Kinetoplastida order and Trypanosomatidae family) together cause life threatening diseases in millions of people as well as wild and domestic animals. The common human diseases caused by these parasites include Chagas disease/American trypanosomiasis by *T*. *cruzi*, sleeping sickness/African trypanosomiasis by *T*. *brucei* spp. and various forms of Leishmaniasis by *Leishmania* spp. Half a billion people are at risk primarily in developing countries in tropical and sub-tropical regions [1]. No vaccines are available and current treatments are inadequate and limited to a few drugs which show toxicity or for which resistance is developed.

These three flagellated protozoans, also known as the “Tritryps”, have a similar genomic organization and a great number of homologous genes in common. They share subcellular structures such as a single flagellum, a single large mitochondrion with a DNA network known as the kinetoplast, and the glycosome, a modified peroxisome. Despite these commonalities, all of these parasites have markedly different life cycle features. *Leishmania* spp are transmitted by sandflies. In the mammalian host promastigotes colonize macrophages and other phagocytic cells and then differentiate into intracellular amastigotes. On the other hand, *T*. *brucei* is transmitted from the salivary glands of the tsetse fly when the insect bites the mammalian host and then lives extracellularly in its vertebrate host. *T*. *cruzi* is transmitted to the vertebrate host in the form of metacyclic trypomastigotes excreted from the hindgut of an insect of the Triatominae subfamily and has both extracellular and intracellular forms (amastigotes). Several morphologically and metabolically distinct forms can be identified in each host with some being replicative and others being non-replicative. In *T*. *cruzi*, intracellular amastigotes are replicative, whereas bloodstream trypomastigotes are non-replicative and travel in the bloodstream extracellularly [2]. After a blood meal, parasites replicate in the digestive tract of the insect in the epimastigote form, and then differentiate to non-replicative metacyclic trypomastigotes in the rectal ampoule.

Finely regulated transitions between non-replicative and replicative forms necessarily imply the existence of mechanisms to rapidly initiate protein synthesis. Ribosomal components need to be coordinately upregulated, assembled, and exported to the cytoplasm for protein synthesis to occur. In the Tritryps, the large ribosomal subunit contains 25/28S rRNA, 5.8S rRNA, and 5S rRNA. Unlike other eukaryotes, the 25/28S rRNA is cleaved at several positions during maturation to produce two major molecules, α and β, and four smaller species [3, 4]. The small ribosomal subunit contains 18S rRNA. The 25/28S rRNA and the 18S rRNA of these organisms contain unusually large expansion segments [5, 6], providing a potential scaffold for the association of trypanosome-specific factors.

We have previously identified and characterized in *T*. *brucei*, two trypanosome-specific RNA-binding proteins, TbP34 and TbP37, with no homologs outside of the Tritryps [7]. They contain two RNA recognition motifs (RRMs), and they specifically associate with 5S rRNA and the ribosomal protein L5, a constituent of the large subunit [8]. P34 and P37 are almost identical in sequence although P37 has an N-terminal 18 amino acid insertion, absent in P34. Although their localization and biochemical properties are largely identical, P34 and P37 are developmentally regulated, P34 being more abundant in procyclic cells, and P37 more abundant in bloodstream cells [9]. These proteins are essential for the survival of the parasite [10]. In their absence, 5S rRNA is specifically destabilized, and ribosome assembly is compromised. Their specific association with conserved components of the ribosomal pathway is being investigated as a potential chemotherapeutic target, therefore a better understanding of the homolog(s) in *T*. *cruzi* was of interest. Previously published work identified two homologs to TbP34 and TbP37 in *T*. *cruzi* [11]. In this work, we characterize biochemical and cellular features of TcP37/NRBD, a *T*. *cruzi* homolog of TbP34 and TbP37.

## Materials and Methods

### Cloning and expression of recombinant proteins

TcP37/NRBD was amplified from *T*. *cruzi* CL Brener genomic DNA using primers TcP37/NRBDF (5' CACCATGCCCGCCAAGTCTGCCAAC 3') and TcP37/NRBDR (5' TTACTTCGTGTGGTTCTTTCTCTTGTT 3') and cloned into vector pET100D-TOPO (Life Technologies). TcL5 was cloned similarly using primers TcL5F (5' ATGCCATTCGTTAAGGTTGT 3') and TcL5R (5' TTACTTCGACGCACGTTCGC 3'). These plasmids were transformed into BL21 star (DE3) One Shot chemically competent *E*. *coli* cells. Expression of 6×His tagged recombinant proteins was induced with 1 mM IPTG for 4 hours at 37°C. Purification of protein was performed by affinity chromatography using a Ni-NTA column (Qiagen) as previously described [8]. Protein concentration was determined using the Bradford assay. SDS-PAGE followed by Coomassie staining was performed to monitor purification of recombinant proteins.

### Synthesis of radiolabeled 5S rRNA

The full length Tc 5S rRNA gene was amplified from CL Brener genomic DNA using primers Tc5SF (5'ATTAACCCTCACTAAAGGGAG GGTACGACCATACT 3') and Tc5SR (5' AAGGGTACGGCACCCCGGGTTCCAGCGCC 3'). The first 21 nucleotides of the forward primer contain the T3 promoter sequence. The purified PCR product was used as the template for T3 polymerase directed *in vitro* transcription (Maxiscript, Life Technologies) in the presence of [α^32^-P] UTP. The full length radiolabeled transcribed RNA was purified from truncated products and unincorporated nucleotides using NucAway spin columns (Life Technologies) and purification was confirmed by electrophoresis using 10% TBE-Urea gels.

### Filter binding assays

Filter binding assays were performed by incubating increasing concentrations (0 to 500 nM) of purified rTcP37/NRBD and rTcL5 with a constant concentration (0.5 fmol) of internally labeled Tc 5S rRNA in a total volume of 50 μl 1×binding buffer (10 mM Tris pH 7.4, 1 mM EDTA, 100 mM NaCl, 0.1% NP40, 100 μg/mL BSA). The reactions were incubated at room temperature for 30 minutes and then applied onto a nitrocellulose filter prewetted in 1×binding buffer. Protein bound RNA was captured in nitrocellulose filters whereas unbound RNA was captured in nytran filters underneath the nitrocellulose filters using a Bio-Dot Microfiltration Apparatus (Bio-Rad). Following filtration, the membranes were washed twice with 1×binding buffer and left to dry at room temperature. A Bio-Rad phosphorimager was used to measure radioactivity associated with both bound and free RNA and Quantity One software was used to quantify the results. Graphpad Prism 5 was used to calculate the dissociation constant (K_d_) by the best fit to a nonlinear regression curve. Binding assays were performed three times with different preparation of recombinant proteins and 5S rRNA.

### Cell culture

*T*. *cruzi* strain CL Brener (kindly provided by Dr. Docampo, University of Georgia) was used throughout this study. Epimastigote forms were maintained at 28°C in liver infusion tryptose medium [12] (5 g/L liver infusion, 5 g/L bactotryptose, 68 mM NaCl, 5.3 mM KCl, 22 mM Na_2_PO_4_, 2 g/L glucose, 0.02 g/L hemin) supplemented with 10% heat inactivated fetal bovine serum, 100 mg/L streptomycin and 100 units/ml penicillin.

### Immunoprecipitation (IP) and immunoblot

Whole cell extracts for co-immunoprecipitation were prepared as previously described [13]. Briefly, 1×10^10^ cells were washed in PBS three times and resuspended in hypotonic buffer (20 mM Tris-HCl pH 7.4, 2 mM EDTA, 1% Triton X-100 and one tablet of protease inhibitor cocktail (Roche) per 5 ml of buffer) followed by three freeze-thaw cycles in liquid nitrogen and in a 42°C waterbath respectively. Cell lysates were then sedimented at 16,000×g for 30 minutes at 4°C. The supernatant was used for IP as previously described [14]. A goat affinity-purified antibody raised against a C- terminal peptide of the TcL5 (CKEKIAFLVASIRERASK, Bethyl Laboratories) and anti-TbP34/P37 antibodies [7] were cross-linked to Protein A labeled magnetic beads (Dynabead, Life Technologies). Antibody coated beads were incubated with 1 mg cell extract at 4°C overnight. The supernatant was removed and the beads coated with antibody-antigen complexes were washed in PBS-T three times followed by elution of protein complexes from the beads by resuspension in SDS-PAGE sample buffer and incubation at 70°C for 10 minutes. Finally, the eluted fractions and the supernatants were analyzed by 12% SDS-PAGE followed by western analysis using antibodies directed against TcL5 and TbP34/P37 as previously described [8]. Cell extracts were incubated with 20 U RNase A (Life Technologies)/ ml extract for 1 hour at 37°C.

To perform IP with recombinant proteins, 500 ng of rTcP37/NRBD were incubated with an equal amount of *in vitro* transcribed Tc 5S rRNA (synthesized as described above using unlabeled UTP) for 10 minutes. Five hundred ng of rTcL5 were added and then the mixture was applied to TcL5 antibody cross-linked Dynabeads. Finally, the eluate and the supernatant fractions were subjected to western analysis using P34/P37 antibodies. These experiments were repeated three times and representative blots are shown.

### Sequential RNA immunoprecipitation

RNA immunoprecipitations were performed using antibodies against TbP34/P37 and TcL5. Approximately 0.15 μg/μL poly dI-dC (deoxy-inosinic deoxy-cytidilic acid, Sigma) was incubated with TbP34/P37 antibody crosslinked Dynabeads (Life Technologies) to reduce nonspecific RNA binding. Two mg of whole cell extract were incubated with Dynabeads at 4°C overnight. The beads were washed in PBS-T (0.05% Tween) three times and eluted by resuspension in elution buffer (1% SDS, 50 mM DTT and 10% β- mercaptoethanol) followed by incubating at 70°C for 10 minutes. Samples were diluted in PBS to an SDS concentration of 0.03%, allowed to renature at room temperature and subjected to a second round of IP as described above but with Dynabeads coated with the anti-TcL5 antibody. Following this second round of IP, bound RNA in the eluted complexes were extracted using Trizol LS (Life Technologies). Subsequent reverse transcript PCR was performed using primers specific for 5S rRNA. Amplified products were electrophoresed in 2% agarose gel and visualized by ethidium bromide staining on a UV transilluminator. The experiments were performed in triplicate and a representative result is shown.

### Binding to poly (A)^+^ RNA

Binding of TcP37/NRBD to poly (A)^+^ RNA was examined as previously described [15], with some modifications. Briefly, 3×10^9^ epimastigote cells at a density of 2–4×10^7^ were used in each experiment. Cells were washed twice and then resuspended in 1×PBS and placed in 100mm×15mm polystyrene petri dishes. Protein-RNA complexes were crosslinked by UV illumination (UV Stratalinker 2400, Stratagene). Cells were sedimented in hypotonic buffer, homogenized in a Dounce homogenizer and passed through a 25 gauge needle. Cell lysates were applied to Dynabeads Oligo(dT) reagent (Life Technologies) and poly (A^+^) fractions were eluted according to the manufacturer’s instructions. Lysate, supernatant, wash and eluate fractions were subjected to western analysis using antibodies against TbP34/P37 and TbPABP1 (Poly-A binding protein-1) [16]. To enhance detection in the western analysis, we used Super Signal West Femto Chemiluminescent substrate (Thermo Scientific). The experiments were performed in triplicate and a representative result is shown.

### Indirect fluorescence microscopy

Immunofluorescence microscopy was performed as previously described [17] using antibodies directed against TbP34/P37 [7] and TbL1C6 [18] at 1:200 dilutions. Secondary goat anti-mouse Alexa Fluor 594 or goat anti-rabbit Alexa Fluor 488 (Life Technologies) were used at 1: 1000 dilutions. Cells were stained in Prolong Gold Antifade reagent with DAPI (Life Technologies). Subcellular localization was analyzed using a Zeiss Axioimager M2 Microscope and the Velocity 6.1 Acquisition software. The experiments were performed in triplicate and a representative result is shown.

#### Subcellular fractionation

Nuclear and cytoplasmic extracts were prepared as described previously [19]. Methanol precipitation was used to recover proteins from the extracts for subsequent western analyses using antibodies against the cytosolic marker PGK (phosphoglycerate kinase) [20], the nuclear marker NOG1 (nucleolar G protein 1) [21] and P34/P37. The experiments were performed in triplicate and a representative result is shown.

## Results

### TcP37/NRBD is an RRM-containing homolog of *T brucei* TbP34/P37

Previous work in *Trypanosoma cruzi* CL Brener by Gomes at al [11] identified homologs to TbP34 and TbP37, which they named TcRRM1 and TcRRM2. These genes are not present in the TritrypDB but are present in Genbank as accession numbers AY590473 and AY590474, respectively. The Trytryp database contains two homologous genes for CL Brener [22] (designated TcCLB.511727.270 and TcCLB.511727.290). Examination of the *T*. *cruzi* strains present in the Tritryp database (S1 Fig) showed that there are either one or two homologous genes in each strain with some variability in the 5’ sequence encoding the N-terminal APK rich domain, a region of low complexity.

**Fig 1:**
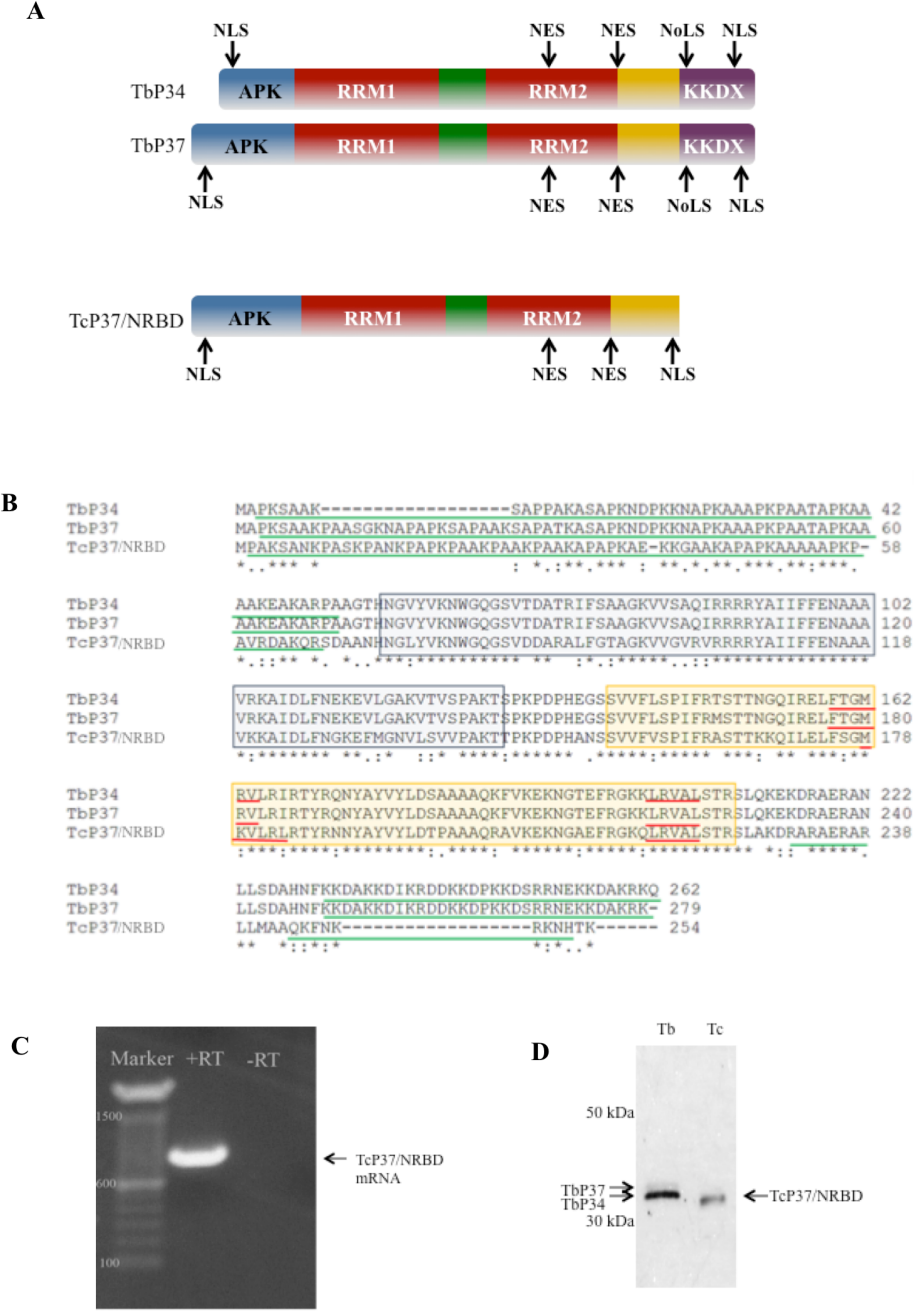
TcP37/NRBD is a homolog of TbP34 and TbP37. **Panel A.** Schematic diagram of TbP34, TbP37, and TcP37/NRBD. APK: alanine-proline-lysine rich region. RRM: RNA Recognition Motif. KKDX: Region containing repeats of lysine, lysine, aspartic acid, and X representing any amino acid. NES: Nuclear Export Signal. NLS: Nuclear Localization Signal. NoLS: Nucleolar Localization Signal. **Panel B.** Multiple sequence alignment of TbP34, TbP37 and TcP37/NRBD protein. An asterisk indicates identity, a colon indicates conserved substitution, and a period indicates semi-conserved substitution. The amino acids of the proteins are indicated by terminal numbers. Blue and yellow boxes indicate RRM1 and RRM2 domains respectively, solid red underlines denote the predicted nuclear export signal, and solid green underlines indicate predicted nuclear localization signals of these proteins. **Panel C.** TcP37/NRBD is endogenously expressed as mature mRNA in epimastigotes. RT-PCR was performed on *T*. *cruzi* total RNA. RT: control with no reverse transcriptase, +RT: amplified specific product, Marker: DNA molecular weight markers (bp). **Panel D.** Western blot analysis of 2 micrograms of whole cell extract protein prepared from *T brucei* procyclic cells (Tb) and *T*. *cruzi* epimastigote cells (Tc). The membrane was probed using the P34/P37 antibody. Two bands are visible in the Tb lane (TbP34 and TbP37), and a single band is observed for Tc (TcP37/NRBD). Numbers indicate molecular weight markers.

In order to characterize the *T*. *cruzi* homologs to TbP34 and TbP37, we designed two sets of primers that would amplify the genes for both TcRRM1 and TcRRM2 [11] or the homologous genes in the Tritryp database [22]. Using genomic DNA from a *T*. *cruzi* CL Brener strain, we obtained PCR products and cloned them into pTrcHis (Life Technologies). We consistently obtained a single RBP homologous gene sequence, which shares 94% and 95% identity with the genes encoding TcRRM1 and TcRRM2 and 94% and 91% identity with TcCLB.511727.270 and TcCLB.511727.290, respectively (BLAST analysis). This gene (Genbank accession number AF316151) had been previously annotated in the *T*. *cruzi* Tulahen strain [23], and was present in the CL Brener whole genome shotgun reads [24]. Like all of the *T*. *cruzi* and *Leishmania* homologous genes, this gene is truncated at the 3’ end encoding the C-terminal end relative to the *T*. *brucei* genes. The protein predicted from the gene we amplified will be called TcP37/NRBD, based on the presence of an N-terminal 18 amino acid stretch (present in TbP37 and absent from TbP34) (Fig 1, Panel A and B). However, the calculated molecular weight of TcP37/NRBD (27.2 kDa) is closer to that of TbP34 (28.8 kDa) than TbP37 (30.3 kDa), because TcP37/NRBD has a shorter C-terminal domain. TbP34 and TbP37 are also identified in the Tritryp database as NRBD1 (nuclear RNA binding domain 1) and NRBD2 respectively. On the basis of this nomenclature we propose TcNRBD2 as an alternative name for TcP37/NRBD [25]. TcP37/NRBD is 59% and 61% identical to TbP34 and TbP37, respectively by global sequence alignment analysis. We conducted phylogenetic analysis of TcP37/NRBD and its available homologs in the Tritryp database, as well as the sequences from the Gomes study [11](S1 Fig). All of the *T*. *cruzi* sequences form a cluster and are highly related to each other whether there are one or two genes per strain. Further *in silico* analysis showed that TcP37/NRBD has an isoelectric point (pI) of 11.06, higher than that of TbP34 (10.49) and TbP37 (10.53). Like TbP34 and TbP37, TcP37/NRBD also has two RNA recognition motifs (RRMs, positions 73 to 143 and 154 to 225) (Fig 1, Panel A and B).

We next analyzed whether TcP37/NRBD was expressed as mature mRNA. We used an oligo(dT) primer to generate cDNA by reverse transcription from whole cell RNA and then amplified this cDNA with primers specific for TcP37/NRBD (nucleotide positions 1 to 20 for forward primer and 736 to 762 for reverse primer). We detected a PCR product of the expected size, 756 bp (Fig 1, Panel C) that was absent when reverse transcriptase was omitted from the reaction. This strengthens the conclusion that TcP37/NRBD is the only TbP34/P37 homolog expressed as mature mRNA in our *T*. *cruzi* CL Brener strain. In addition, we purified this PCR product from the agarose gel and sequenced it directly, confirming that it indeed encoded TcP37/NRBD.

To examine expression at the protein level, we performed western blot analysis on whole cell extracts of *T*. *brucei* and *T*. *cruzi* using the P34/P37 antibody. Our results (Fig 1, Panel D) show that, while both TbP34 and TbP37 are detected in the *T*. *brucei* cell extract as expected, only one band is detectable in the *T*. *cruzi* lane, confirming our findings that this *T*. *cruzi* strain expresses TcP37/NRBD. Previous data from Gomes *et al* using the same antibodies [11] showed two distinct bands expressed representing TcRRM1 and TcRRM2. Quantitative proteomic data [26] demonstrated expression of a homolog (TcCLB.511727.270 but no data for TcCLB.511727.290) for *T*. *cruzi* strain Dm28c.

### Analysis of potential localization signals

TbP34 and TbP37 both have a classical bipartite lysine-rich nuclear localization signal (NLS). A classical NLS consists of two stretches of basic amino acids separated by a linker region of 9 to 12 amino acid residues [27]. In TbP34 and TbP37 this signal comprises the last 16 amino acids and it has the sequence ^247^ **KK**DSRRNEKKDA**KRK** ^261^ (TbP34 numbering, basic residues in bold). However, because part of this signal is found in the C-terminal domain, TcP37/NRBD (and the other *T*. cruzi homologs) does not appear to possess a classical bipartite NLS. The algorithm NucPred [28], which predicts nuclear localization of proteins, provides a score of 0.40 for TcP37/NRBD and a score of 0.61 (higher scores mean higher confidence for nuclear localization) for both TbP34 and TbP37 (Table 1). These scores reflect a likelihood of nuclear localization since 63% and 71% of all proteins scoring 0.40 and 0.60 respectively are in fact nuclear. In addition, 69% and 53% of bona fide nuclear proteins will score above 0.40 and 0.60 respectively using this algorithm. In addition, the HMM-based algorithm NLStradamus identifies sequences in TbP34, TbP37 and TcP37/NRBD with nuclear localization signals in both N- and C- terminal regions (Table 1, Fig 1 Panel A and B). TbP34 and TbP37 also have nuclear export signals (NES) and these proteins have been experimentally validated as factors involved in the export of ribosomal subunits [29]. Based on prediction by the NetNES algorithm [30], TcP37/NRBD also possesses a nuclear export signal (Fig 1, Panel B and Table 1). Another algorithm ValidNES [31] also predicts NES for all of these proteins (Table 1, Fig 1 Panel A and B). The algorithm NoD [32], which predicts nucleolar localization signals (NoLS), detects no NoLS in TcP37/NRBD, but it predicts a NoLS both for TbP34 and TbP37 in the C-terminal region which is absent from TcP37/NRBD. However, Nuc-PLoc [33], a subnuclear localization predicting server, predicts TbP34, TbP37, and TcP37/NRBD to all have nucleolar localization.

**Table 1:** Localization signals of TbP34, TbP37 and TcP37/NRBD predicted by the indicated algorithms.

Proteins	Localization Signals	Algorithms
	**NLS**	
TbP34	PKSAAKSAPPAKASAPKNDPKKNAPKAAAPKPAATAPKAAAAKEAKARPA	**NLStradamus**
TbP34	KKDAKKDIKRDDKKDPKKDSRRNEKKDAKRKQ	**NLStradamus**
TbP37	PKSAAKPAASGKNAPAPKSAPAAKSAPATKASAPKNDPKKNAPKAAAPKPAATAPKAAAAKEAKARPA	**NLStradamus**
TbP37	KKDAKKDIKRDDKKDPKKDSRRNEKKDAKRK	**NLStradamus**
TcP37/NRBD	PAKSANKPASKPANKPAPKPAAKPAAKPAAKAPAPKAEKKGAAKAPAPKAAAAAPKPAVRDAKQR	**NLStradamus**
TcP37/NRBD	RARAERAR	**NLStradamus**
TcP37/NRBD	QKFNKRKNH	**NLStradamus**
TbP34	Score 0.6	**NucPred**
TbP37	Score 0.6	**NucPred**
TcP37/NRBD	Score 0.4	**NucPred**
	**NES**	
TbP34	LRVAL	**NetNES**
TbP37	LRVAL	**NetNES**
TcP37/NRBD	LRVAL	**NetNES**
TbP34	FTGMRV	**ValidNES**
TbP37	FTGMRV	**ValidNES**
TcP37/NRBD	MKVLRL	**ValidNES**
	**NoLS**	
TbP34	SDAHNFKKDAKKDIKRDDKKDPKKDSRRNEKKDAKRKQ	**NoD**
TbP37	SDAHNFKKDAKKDIKRDDKKDPKKDSRRNEKKDAKRKQ	**NoD**
TcP37/NRBD	**-**	**NoD**
TbP34	**+**	**Nuc-PLoc**
TbP37	**+**	**Nuc-PLoc**
TcP37/NRBD	**+**	**Nuc-PLoc**

NLS: Nuclear localization signal, NES: Nuclear export signal, NoLS: Nucleolar localization signal.

### TcP37/NRBD is localized to the nucleolus, nucleus and cytosol

**Fig 2:**
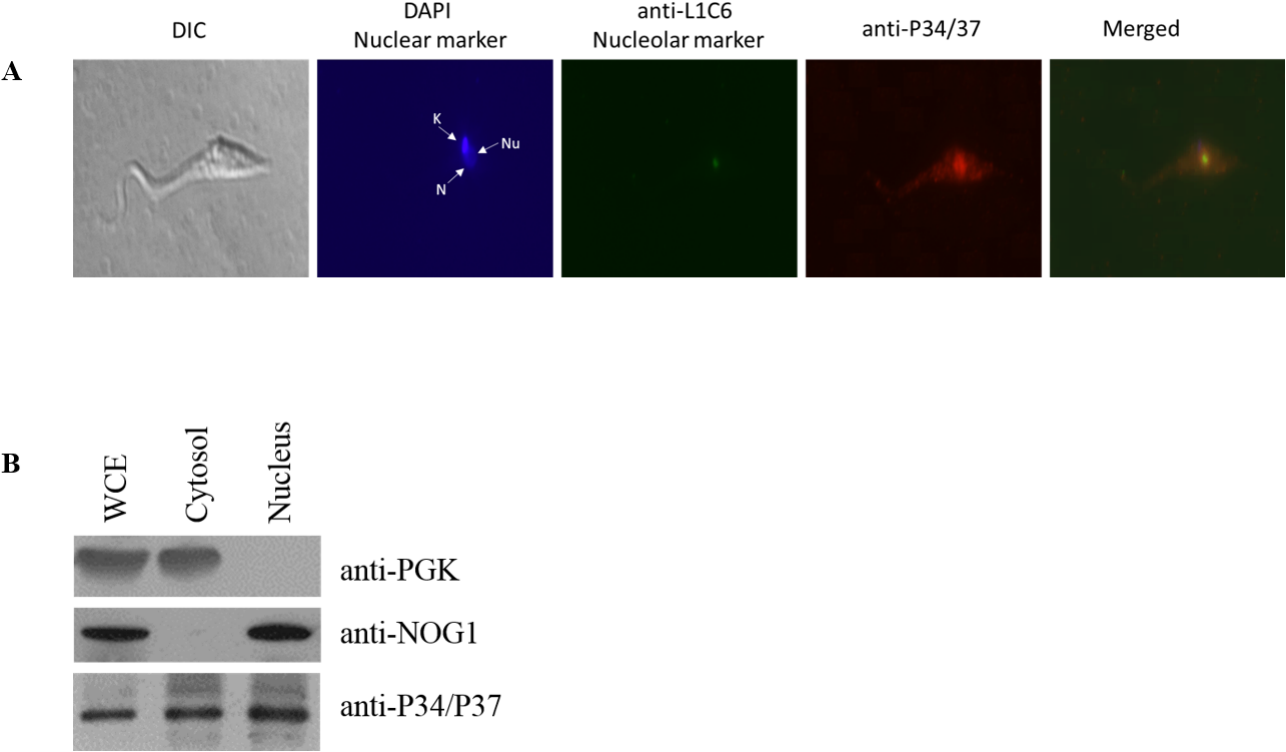
TcP37/NRBD is present in the nucleus, nucleolus, and cytosol. **Panel A.** Immunofluorescence microscopy of epimastigote CL Brener cells. Parasites were stained with a primary antibody directed against TbP34/P37 (red), an antibody against nucleolar marker L1C6 (green), and DAPI (blue) to stain the nucleus and the kinetoplast.The corresponding differential interference contrast (DIC) image is shown on the far left and the merged field on the far right. N: Nucleus, Nu: Nucleolus, K: Kinetoplast. **Panel B.** Western blot analysis of biochemical fractionation of epimastigotes into cytosolic and nuclear fractions. Two micrograms of total protein for each fraction were probed with antibodies against cytoplasmic marker PGK (top panel), nucleolar marker NOG1 (middle panel) and P34/P37 (bottom panel). WCE: Whole cell extract.

In order to clarify the *in silico* predictions, we undertook two different approaches, immunofluorescence analysis and biochemical subcellular fractionation. Immunofluorescence studies of the epimastigote stage of CL Brener cells allowed us to visualize colocalization of the signal obtained using the antibody previously raised against TbP34/P37 with the signal for nuclear (DAPI) and nucleolar (L1C6) markers. We were able to detect the presence of TcP37/NRBD in the nucleus as well as in the cytoplasm (Fig 2, Panel A). This is in agreement with the results from the algorithms used to predict subcellular localization and supports a function for TcP37/NRBD related to its binding to 5S rRNA (a molecule that is synthesized in the nucleus and is transported to the cytoplasm in the large ribosomal subunit). Within the nucleus, TcP37/NRBD colocalizes with the nucleolar marker L1C6, not in agreement with NoD but with Nuc-PLoc predictions. Again, this supports the hypothesis that TcP37/NRBD functions in association with 5S rRNA since it is transcribed in the nucleoplasm, but must be transported to the nucleolus for incorporation into the pre-ribosomal particle.

To confirm the immunofluorescence results, we performed biochemical subcellular fractionation of epimastigote cells. After fractionation, aliquots were analyzed by western blotting using antibodies against a cytosolic marker (PGK), a nuclear marker (NOG1), and P34/P37. As shown in Fig 2, Panel B, PGK is detected in whole cell extracts and cytoplasmic fractions as expected, but is not detectable in the nuclear fraction. Conversely NOG1 is readily detectable in whole cell extracts and nuclear fractions, but not in the cytosolic fraction. Taken together, these results indicate that our cytoplasmic and nuclear fractions were free of cross contamination within the limits of detection of the assay. When these fractions were tested for the presence of TcP37/NRBD using the P34/P37 antibody, we detected signal in both the cytoplasmic and nuclear lanes. Taken together, our immunofluorescence microscopy and biochemical fractionation data demonstrate that TcP37/NRBD is present both in the nucleus and the cytoplasm, and that within the nucleus is clearly present in the nucleolus.

### TcP37/NRBD and TcL5 association is enhanced by RNA

**Fig 3:**
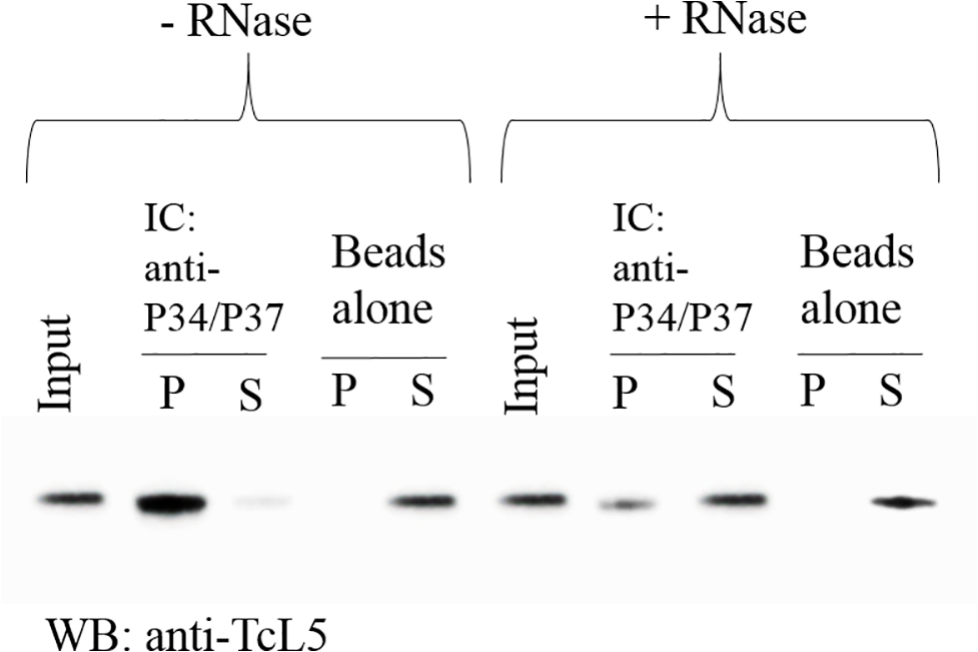
TcP37/NRBD and TcL5 associate in a largely RNA-dependent fashion. Epimastigote whole cell extracts were immune captured with beads cross-linked with anti-P34/P37 antibody. The pellet (P) and supernatant (S) fractions were analyzed by western blot using a anti-TcL5 peptide antibody. The extracts were incubated in the absence (left lanes) or the presence (right lanes) of RNAse A. Beads alone were used as a negative control. Beads alone were used as a negative control. The input lane contains ten percent of the material used for IP.

**Fig 4:**
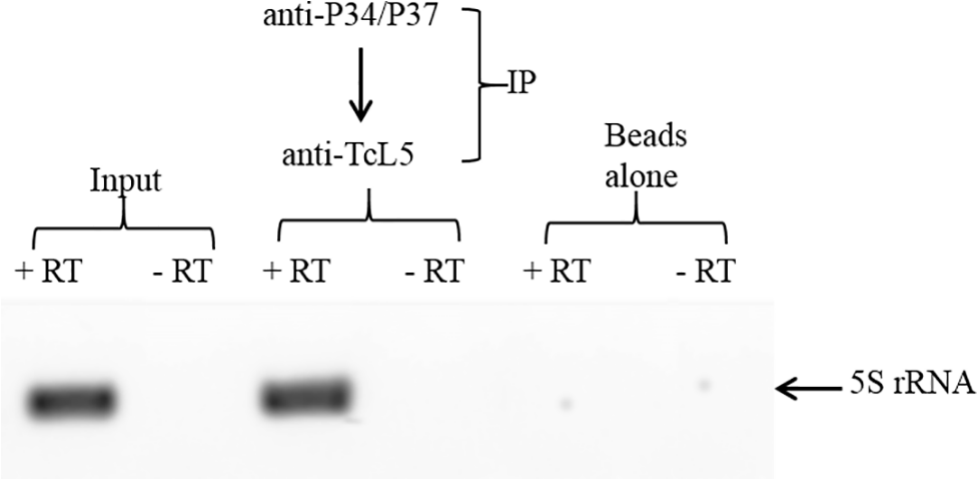
TcP37/NRBD interacts with L5 and 5S rRNA. Whole cell extracts were sequentially immunoprecipitated using an antibody against TbP34/P37 and then an antibody against TcL5 to enrich the precipitate in complexes containing both components. Reverse transcription was then performed on these complexes to specifically detect the presence of 5S rRNA. Beads: beads alone (no antibody),-RT: no reverse transcriptase, +RT: reverse transcriptase included.

We have previously reported an association between two trypanosome specific factors TbP34, and TbP37 with conserved ribosomal protein TbL5 [8]. To investigate whether this novel association exists in *T*. *cruzi*, we performed co-immunoprecipitation experiments using the P34/P37 polyclonal, affinity-purified antibody on whole cell extracts derived from epimastigote form *T*. *cruzi* cells. We then analyzed the immune captured pellet and supernatant fractions by western blot using an antibody against TcL5 peptide. Fig 3 shows that L5 coprecipitates with TbP34 andTbP37 in the pellet (P) fraction. We then investigated whether this association was mediated by RNA by pre-incubating the extracts with RNAse A. As shown on Fig 4, right panel, the RNase A treatment decreases the association between TcP37/NRBD and TcL5 by 74% ±8% (based on three biological replicates). We conclude that TcP37/NRBD associates with TcL5 and this association is strongly enhanced by the presence of RNA.

### TcP37/NRBD forms a complex with 5S rRNA and ribosomal protein L5

5S Ribosomal RNA associates with ribosomal protein L5 as a preribosomal particle as 5S rRNA is being transcribed in the nucleoplasm. In *T*. *brucei*, TbP34 and TbP37 participate in this association, and help stabilize 5S rRNA. Our laboratory has previously shown that the RRMs and the N-terminal motifs of TbP34 participate in the interaction with L5 and 5S rRNA [14, 34]. Given the differences between TcP37/NRBD and TbP34/P37 in the C-terminal domain, it was important to investigate whether TcP37/NRBD could also form a complex with both L5 and 5S rRNA *in vivo*. We used sequential immunoprecipitation to enrich whole cell extracts in complexes containing both TcP37/NRBD and the L5 ribosomal protein. We then tested these complexes for the presence of 5S rRNA by reverse transcription followed by PCR with specific primers. Our results (Fig 4) indicate that the three molecules participate in an association in *T*. *cruzi* epimastigotes, despite the loss of the C-terminal domain in TcP37/NRBD.

### TcP37/NRBD does not associate with messenger RNA

**Fig 5:**
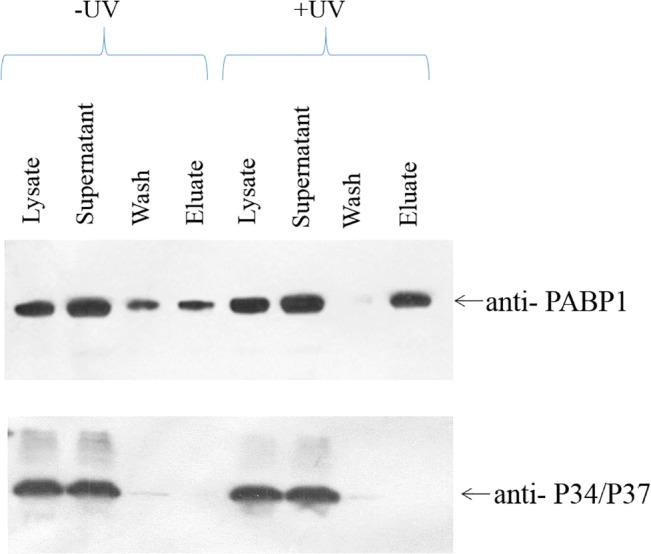
TcP37/NRBD associates with poly(A)- RNA but not with poly(A)+ RNA. Whole epimastigote cell lysates (with and without UV cross-linking) were incubated with oligo(dT) Dynabeads. Fractions were analyzed by western blot using antibodies against P34/P37 and poly(A) RNA binding protein PABP1.

To determine whether TcP37/NRBD has a general RNA binding affinity (lower specificity) or might be involved in binding elements present in other RNA populations, we tested the ability of TcP37/NRBD to associate with the polyadenylated fraction of cellular RNAs (Fig 5). Epimastigote whole cell extracts were incubated with oligo(dT) Dynabeads. After incubation, the beads bound to polyadenylated RNAs and all interacting factors were separated from the supernatant, washed, and finally eluted. These fractions were analyzed by western blot for the presence of PABP1 as control protein known to bind polyadenylated RNAs, and TcP37/NRBD using the P34/P37 antibody. In order to further preserve the RNA-protein complexes, this experiment was also performed with UV crosslinked extracts. Our results show that, as expected, PABP1 associates with the polyadenylated RNA fraction. However, TcP37/NRBD remains in the supernatant and is not bound to the oligo(dT) Dynabeads. Stabilizing the RNA-protein interactions using UV crosslinking of the extracts yielded the same results, indicating that TcP37/NRBD does not interact with polyadenylated mRNAs either stably or transiently.

### TcP37/NRBD and TcL5 bind Tc5S rRNA

**Fig 6:**
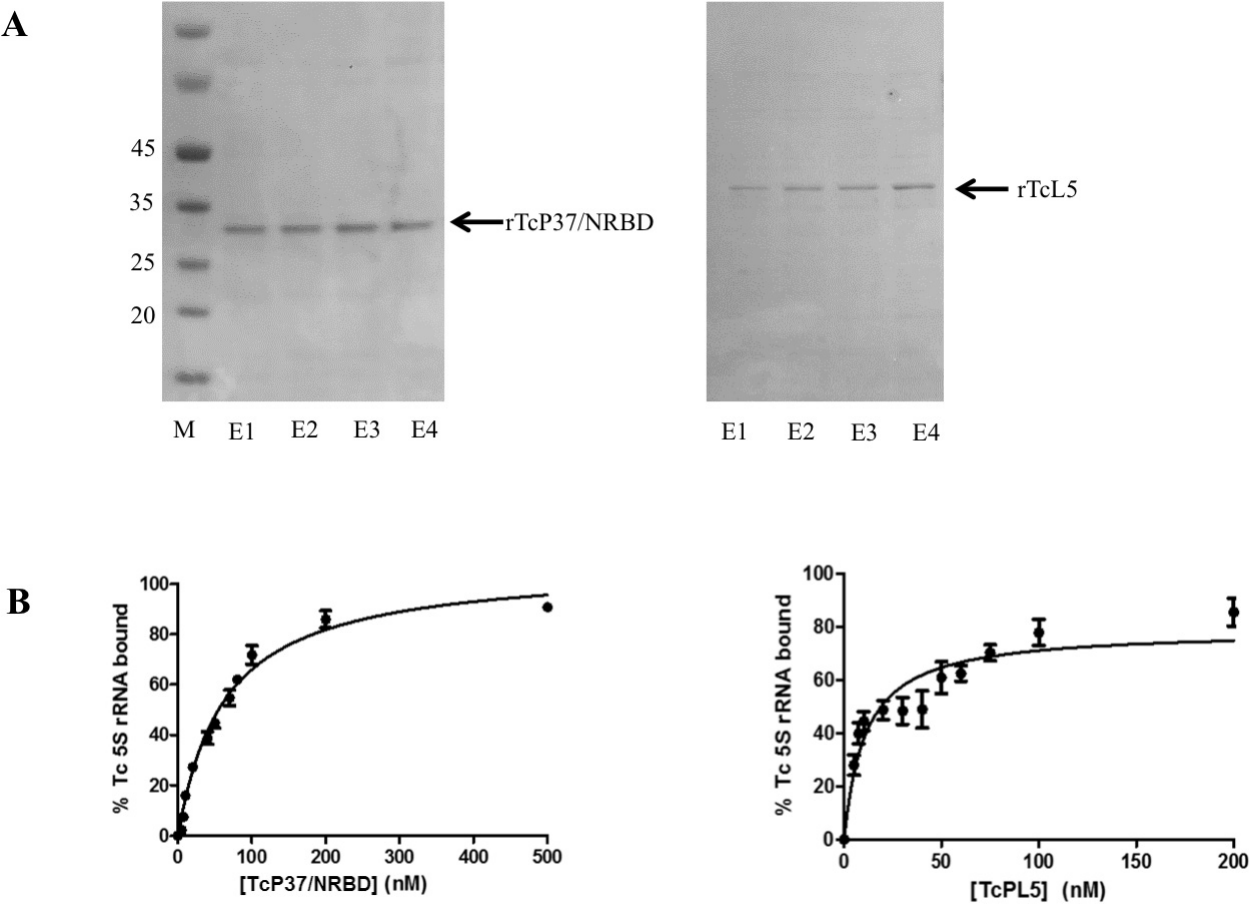
TcP37/NRBD and TcL5 directly bind Tc5S rRNA. **Panel A.** Purification of recombinant TcP37/NRBD and TcL5. Coomassie blue staining of SDS-PAGE of eluted fractions (E1-E4) from a Ni-NTA affinity column. M: Molecular weight markers (in kDa). **Panel B:** Recombinant TcP37/NRBD and TcL5 bind *in vitro* transcribed Tc 5S rRNA. The filter binding assay using radiolabeled *T*. *cruzi* 5S rRNA and rTcP37/NRBD and rTcL5. The percentage of signal bound to the filters was plotted against the concentration of recombinant protein in each reaction. The line represents the fit to a bimolecular equilibration calculated in Graphpad Prism 5.

We have thus far demonstrated an association between TcP37/NRBD and 5S rRNA. This association can be either direct, involving protein-RNA contacts, or indirect, contingent upon other cellular factors. Based on the data previously published on TbP34 and TbP37, we anticipated that TcP37/NRBD would be able to bind 5S rRNA *in vitro*, in the absence of any cellular proteins or RNAs. Therefore, in order to experimentally test this assumption, we purified recombinant TcP37/NRBD (Fig 6, Panel A left) and used it in filter binding assays with radiolabeled, *in vitro* transcribed *T*. *cruzi* 5S rRNA. As shown in Fig 6, Panel B, 90% of the radiolabeled 5S rRNA can be bound by recombinant TcP37/NRBD at saturation levels. The binding data fits a bimolecular equilibrium with a K_d_ value of 63±2 nM. In *T*. *brucei*, TbP34 and TbP37 bind Tb5S rRNA with slightly higher affinity, at 48 and 40 nM, respectively [35].

In addition, we compared the binding affinity of TcP37/NRBD to 5S rRNA to the binding affinity of TcL5 to 5S rRNA. The K_d_ value of eukaryotic L5 ribosomal proteins for 5S rRNA is typically in the low nanomolar range. In the case of *T*. *brucei*, we have previously shown that TbL5 binds 5S rRNA with a K_d_ of 12 nM [8], a value about six-fold higher than the binding constant for *Xenopus* L5 [36, 37], a well-characterized association. After preparation of recombinant TcL5 (Fig 6, Panel A right), we tested the protein obtained in a filter binding assay (Fig 6, Panel B, right). The calculated K_d_ value for this bimolecular binding is 11±3 nM. Therefore, TcL5 has a higher affinity for 5S rRNA than TcP37/NRBD. As in the case of TbL5, the affinity of TcL5 for 5S rRNA is lower than the typical eukaryotic value.

### TcP37/NRBD and TcL5 interact directly in an association that can be enhanced by 5S rRNA

**Fig 7:**
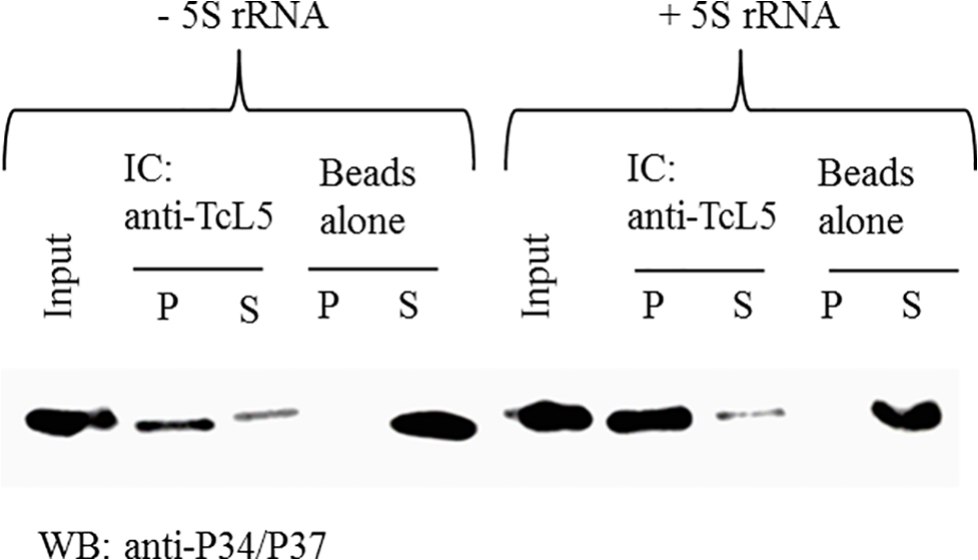
TcP37/NRBD associates with L5 directly and the interaction is modulated by 5S rRNA. rTcP37/NRBD was incubated with rTcL5 in the absence (left lanes) or the presence (right lanes) of *in vitro* transcribed 5S rRNA, and then precipitated with the anti-TcL5 antibody. Western blot analysis of the immunoprecipitate (P) and supernatant (S) fractions was performed using the anti-P34/P37 antibody. Beads alone were used as controls for non-specific interactions with the beads.

To further understand the molecular interactions involving TcP37/NRBD, we asked whether or not TcP37/NRBD is capable to bind directly to TcL5 in the absence of other factors. Recombinant proteins were used in coimmunoprecipitation experiments. After incubation of rTcP37/NRBD with rTcL5, beads covalently attached to the anti-TcL5 peptide antibody were used to pull down rTcL5 and rTcL5 containing complexes. The pelleted or bound fraction (P) and the supernatant or unbound fraction (S) were analyzed for the presence of rTcP37/NRBD. If both factors interact directly, the bound fraction should contain rTcP37/NRBD. Beads alone were used as a control for non-specific interactions. As shown in Fig 7, our results demonstrated that TcP37/NRBD and TcL5 associate directly *in vitro* (compare the P lane in the anti-TcL5 IC with the P lane in the beads control). In addition, we tested the effect of 5S rRNA in the formation of this complex. As shown in Fig 7, the presence of 5S rRNA in the reaction increases the association between TcP37/NRBD and TcL5 by 62±9%. This suggests that the affinity of TcP37/NRBD for TcL5 increases in the presence of 5S rRNA, or that the complex is more stable in the presence of 5S rRNA supporting our results from cell extracts (Fig 3).

## Discussion

Ribosomes, large ribonucleoprotein complexes, are responsible for protein synthesis and therefore essential across all domains of life. Most aspects of the biogenesis of ribosomes are evolutionarily conserved. This process has been extensively studied in yeast but less so in *Xenopus*, mammals and trypanosomes. Although trypanosomes show conserved characteristics in the process [19, 29], some peculiarities have also been reported [5]. Previously, our laboratory has shown the involvement of two trypanosome specific proteins TbP34 and TbP37 in multiple steps of the ribosomal biogenesis pathway in *T*. *brucei* [29]. These proteins form a trimolecular complex with large ribosomal protein L5 and 5S rRNA and stabilize the complex for further incorporation of 5S rRNA into the 90S preribosomal complex [8]. In this study, we identified and characterized TcP37/NRBD, the homolog of P34 and P37 in *T*. *cruzi*. We have determined that the gene for this homolog is actively transcribed and translated into protein in our epimastigote CL Brener cells (Fig 1).

TbP34 and TbP37 localize both to the nucleus and the cytoplasm and possess clear nuclear localization signals in the N-terminal and the C-terminal domains. However, the C terminus of TcP37/NRBD is truncated disrupting the bipartite NLS (Table 1, and Fig 1, Panel B). Nevertheless, other nuclear localization signals are predicted in the N-terminal domain of TcP37/NRBD that might support nuclear localization. Our studies show that despite the loss of the C-terminal domain, TcP37/NRBD does localize to the nucleus, nucleolus, and cytoplasm (Fig 2). In this work we demonstrate that TcP37/NRBD associates with both TcL5 and Tc5S rRNA. Its putative role in the preribosomal complex would support localization in both the nucleoplasm (formation of the preribosomal complex) and in the nucleolus (where the preribosomal complex associates with the assembling 90S complex). We have previously demonstrated that TbP34 and TbP37 bind nuclear export factors Nmd3/Xpo1 [29] and are required for the assembly of the export complex onto the nuclear pre-60S subunit. As part of the 60S biogenesis pathway TcP37/NRBD might also be involved in nuclear export of the 60S ribosomal subunits, explaining its presence in the cytoplasm.

It is also possible that this dual localization might play a role in 5S rRNA shuttling. In *Xenopus* oocytes, the shuttling of 5S rRNA binding proteins is necessary for rapid initiation of cell division. These cells synthesize 5S rRNA in excess amounts during oogenesis and shuttle it to the cytoplasm bound to TFIIIA in the form of a storage particle [38]. The nuclear localization signal of TFIIIA is masked upon binding to 5S rRNA, ensuring cytoplasm localization of this storage particle. When the metabolic needs of the cell increase and ribosomes need to be synthesized in large amounts, L5 displaces TFIIIA and the L5-5S RNP is translocated back to the nucleus, where it is incorporated onto nascent ribosomes [39]. Since TFIIIA is not represented in the Tritryp database, it is possible that TcP37/NRBD and the homologs function in the same role. This possibility requires further exploration.

We have experimentally confirmed that TcP37/NRBD is a 5S rRNA binding factor (Fig 6, Panel B). The dissociation constant is slightly higher for TcP37/NRBD (63 nM) than for TbP34 (48 nM) and TbP37 (40 nM), indicating a slightly lower affinity for 5S rRNA. This correlates with experiments currently underway in the laboratory showing that deletion of the C-terminus of TbP34 leads to a decreased affinity for 5S rRNA (A. Kamina, unpublished data). The C-terminus of RRM containing proteins can increase the RNA binding affinity by increasing the RNA-protein interaction network [40]. For example, C-terminal regions of small nuclear ribonucleoprotein, U1A, and polypyrimidine tract binding protein, PTB, both RRM proteins, directly recognize target RNA molecules and enhance RNA binding affinity [41, 42]. Although the affinity of TcP37/NRBD is decreased for 5S rRNA, it does not bind RNA non-specifically. Polyadenylated RNAs are not associated with TcP37/NRBD (Fig 5), so a direct role in mRNA processing, stability, or localization can be eliminated.

We investigated the ability of TcP37/NRBD to associate with *T*. *cruzi* ribosomal L5 protein and discovered that the two proteins associate with each other in a complex (Fig 3). This association is strongly dependent on the presence of RNA, since treatment of the extracts with RNase A decreased coprecipitation of the proteins. This differs from our results with the *T*. *brucei* proteins where RNAse A treatment had almost no effect on association of TbP34 and TbP37 and TbL5 in cell extracts [8]. The enhanced association here suggests that different motifs in 5S rRNA bind to TcP37/NRBD and to the L5 ribosomal protein and that the interaction of one increases the interaction of the other. The secondary structure of 5S rRNA consists of two arms arranged in five stems (I through V) and five loops (A through E) [38]. In *T*. *cruzi*, 5S rRNA is 98% identical to Tb5S rRNA and folds in the typical structure according to mfold. We have previously shown that loop A/stem V region of the molecule is important for binding to TbP34 [35]. TbL5 has been shown to bind 5S rRNA mainly via the loop C domain [36]. A similar mode of binding may allow 5S rRNA to bind both TcL5 and TcP37/NRBD at the same time, therefore explaining our observation that treatment with RNase partially abolishes the interaction. The remainder of the interaction can be explained by a direct protein-protein association between TcP37/NRBD and TcL5 perhaps via the α helices of the RRMs. In fact, our *in vitro* results with recombinant proteins confirm that TcP37/NRBD can directly interact with TcL5. 5S rRNA enhances the association between the recombinant TcP37/NRBD and TcL5 (Fig 7), in a more pronounced manner than what was previously described for TbP34. Interestingly, no enhancement was seen for the interaction of TbP37 and Tb5S rRNA. Conformation changes of TcP37/NRBD upon binding to 5S rRNA may result in a higher affinity for TcL5 within the trimolecular complex. We have also experimentally determined that TcL5 binds 5S rRNA directly, with a K_d_ value of 11 nM (Fig 6, Panel B right). This value is close to the 12 nM previously reported for TbL5. These L5 proteins share 83% sequence identity. Since TcL5 binds 5S rRNA directly, an alternative explanation for the enhancement of the association between TcL5 and TcP37/NRBD in the presence of 5S rRNA is that a conformational change takes place in TcL5 upon binding of 5S rRNA, increasing its affinity for TcP37/NRBD. Experiments under way in our laboratory will precisely define the contributions of important residues in the RRMs of TbP34 and TbP37 to the binding of L5 and 5S rRNA.

The identification of a homolog of essential trypanosome-specific proteins in *T*. *cruzi* suggests that the Tritryps evolved a unique addition to the ribosomal biogenesis pathway. The Tritryp database also shows that *Leishmania* spp possess either one or two homologs for these proteins which have the same basic structure but like *T*. *cruzi* lack the C-terminal domain of the *T*. *brucei* proteins. Differences in the role of 5S rRNA in the interaction of the two protein components and in the affinity of the homologs for 5S rRNA suggest that the C-terminal domain plays a role in TbP34 and TbP37 interactions that is not seen for the TcP37/NRBD. However, despite significant differences in the C terminal structure of the *T*. brucei and *T*. *cruzi* proteins, we have shown here that the biochemical (binding partners) and cellular biology (expression, localization) characteristics of this factor are largely conserved between *T*. *cruzi* and *T*. *brucei*. We are developing a high throughput system to find molecules that specifically disrupt the essential interactions between trypanosome-specific P34/P37 and conserved components L5/5S rRNA The data we present here indicates that the same molecules could be used to disrupt interactions in the (TcP37/NRBD)/TcL5/Tc5S rRNA complex.

## Supporting Information

S1 Fig Multiple sequence alignment at the protein level of homologs of TcP37/NRBD and Phylogenetic tree reconstructed based on the alignment. (PPTX)
