# Supplementary material for: Kinetoplastid Specific RNA-Protein Interactions in Trypanosoma cruzi Ribosome Biogenesis
Source: PLoS One. 2015 Jun 29;10(6):e0131323. doi: 10.1371/journal.pone.0131323 (PMC4488245; doi:10.1371/journal.pone.0131323)
Supplement: S1 Fig — (PPTX) [file pone.0131323.s001.pptx]

## Slide 1
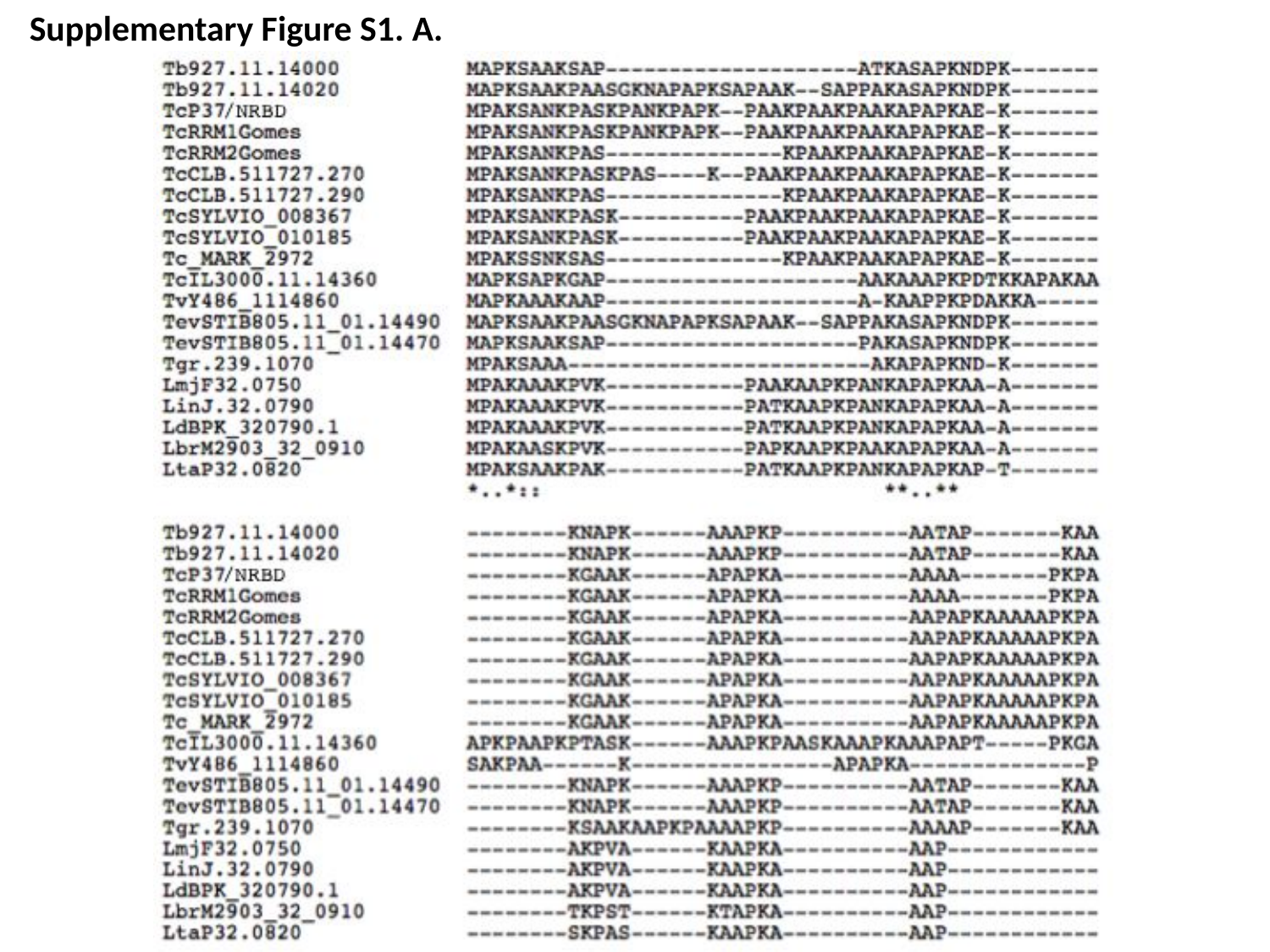

Supplementary Figure S1. A.

## Slide 2
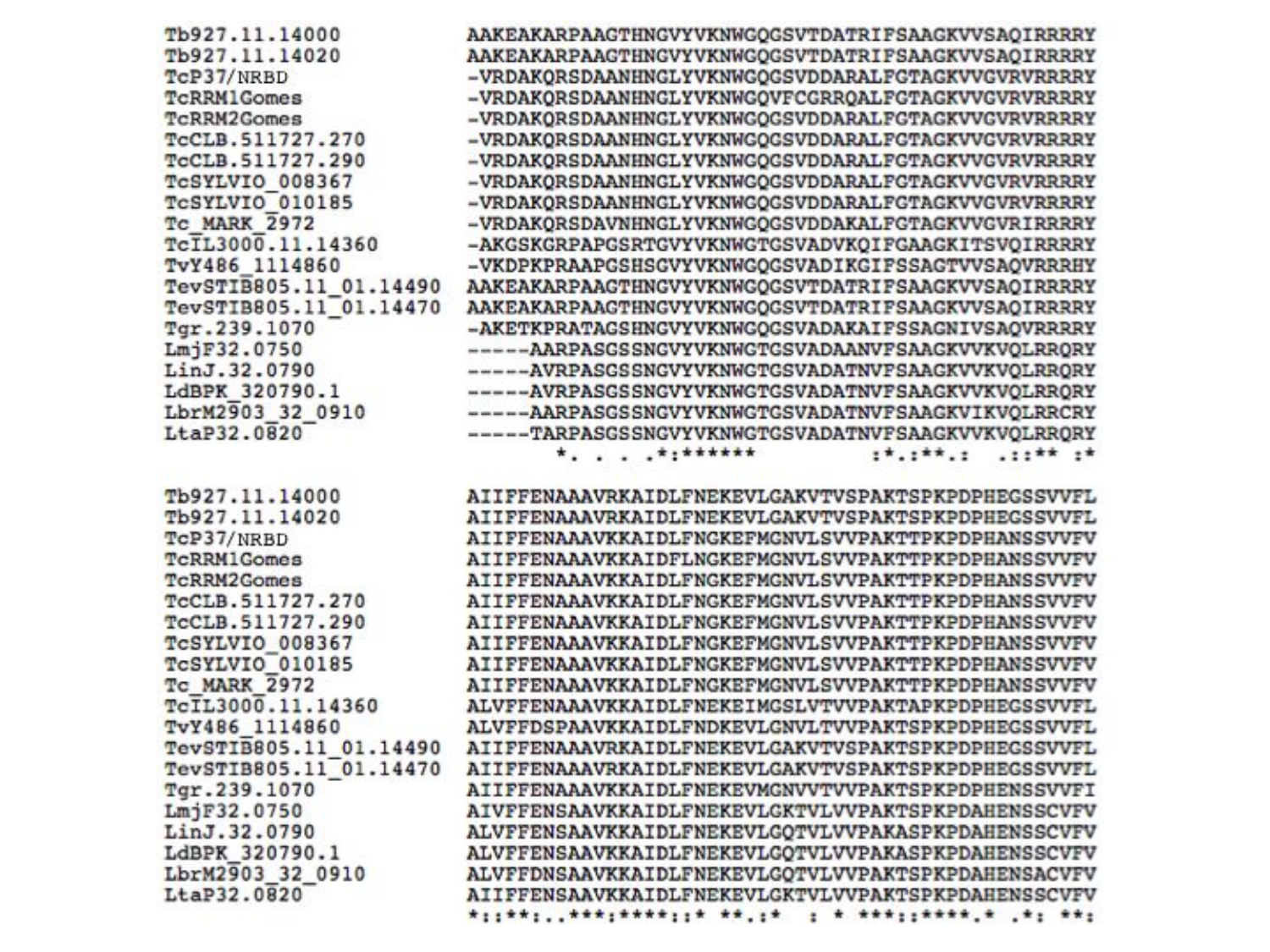

## Slide 3
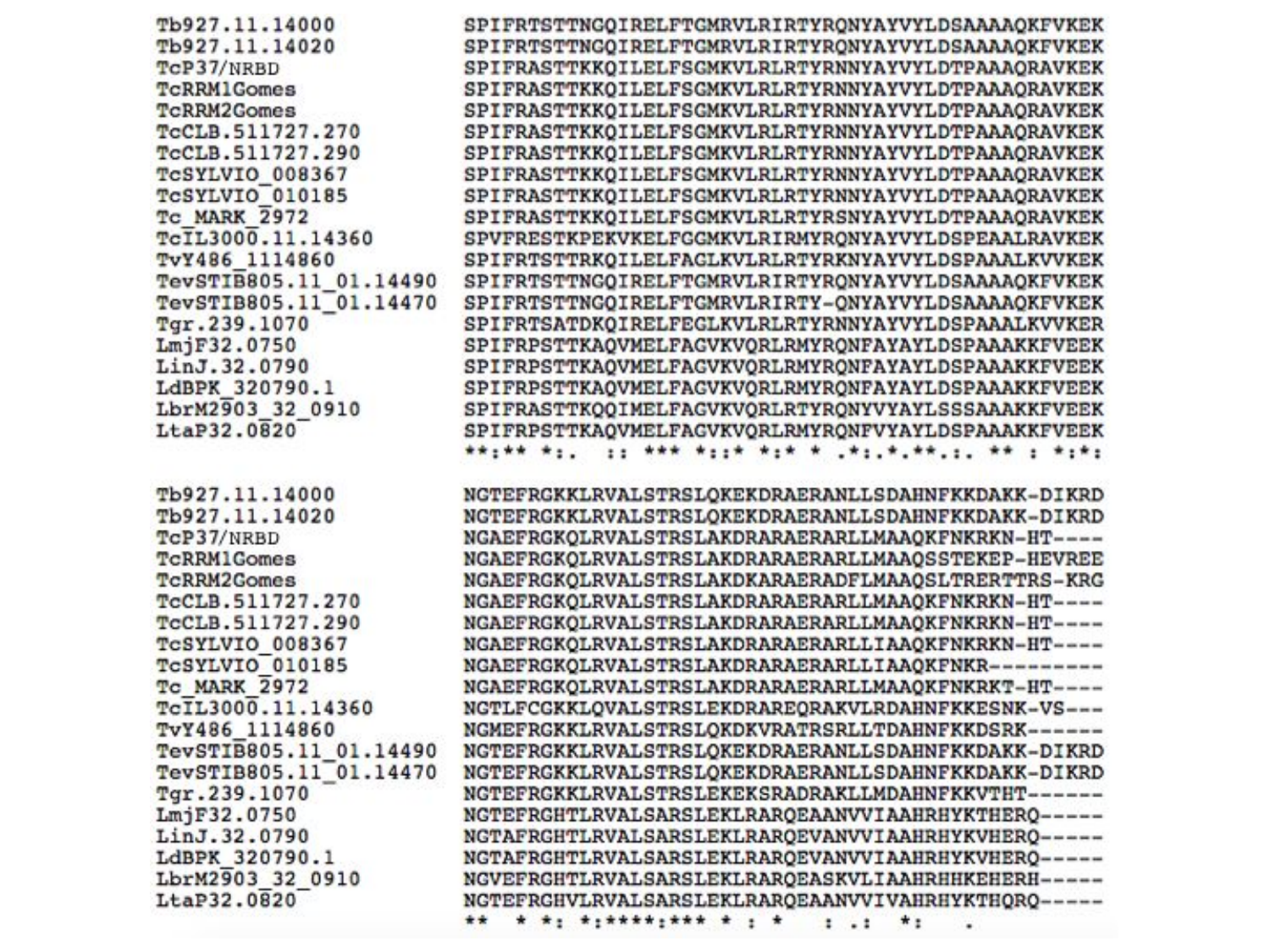

## Slide 4
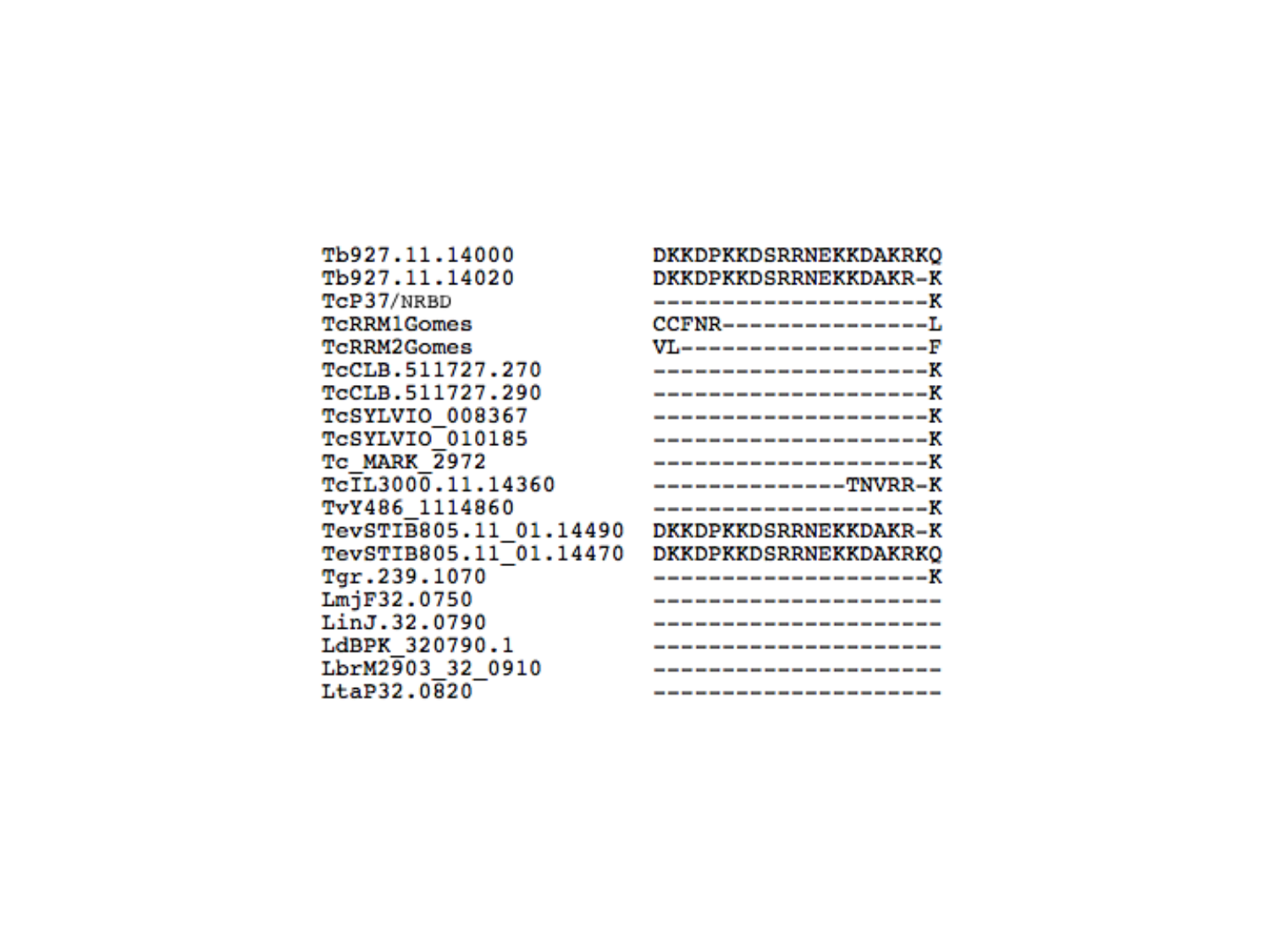

## Slide 5
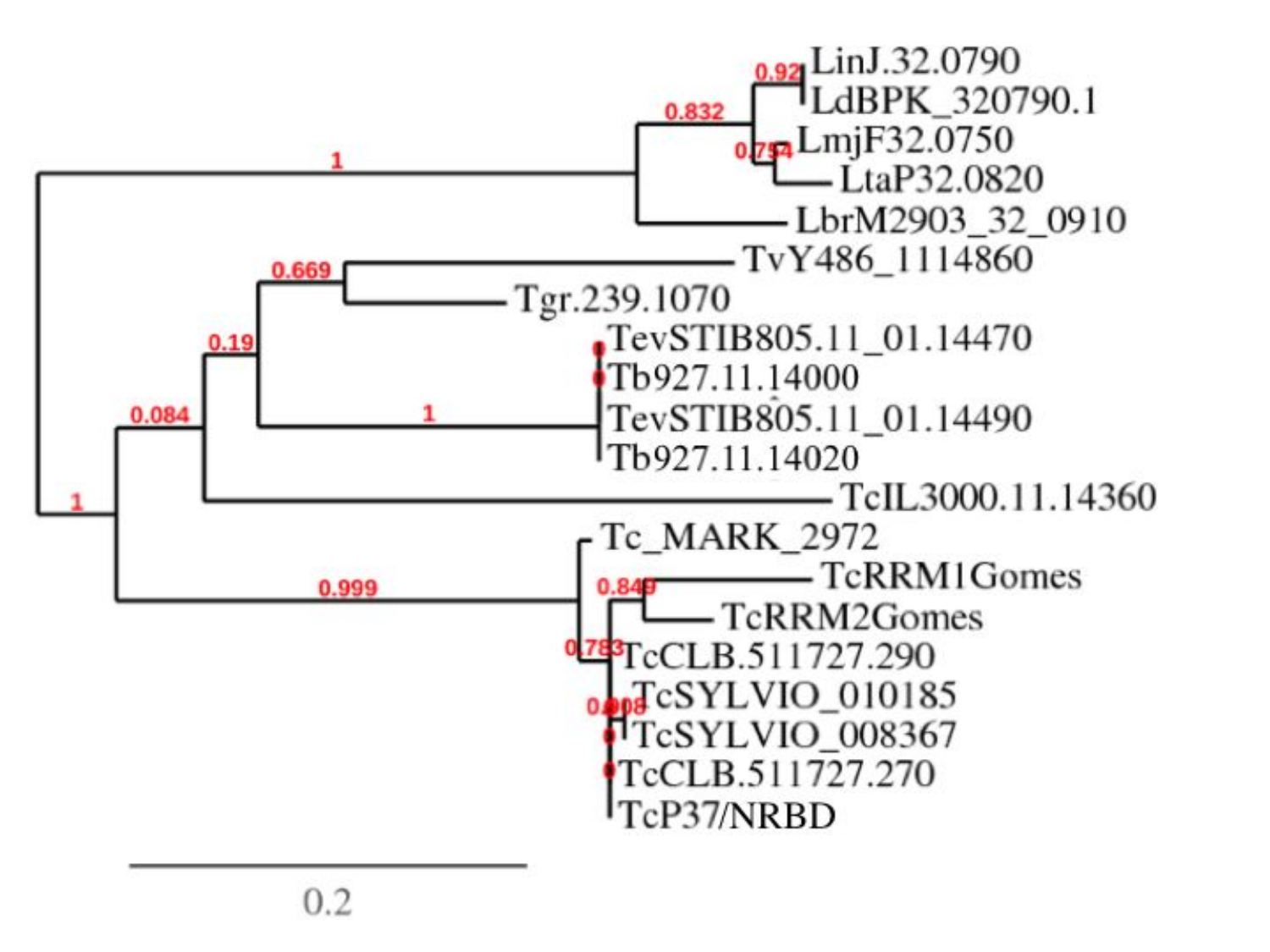

## Slide 6
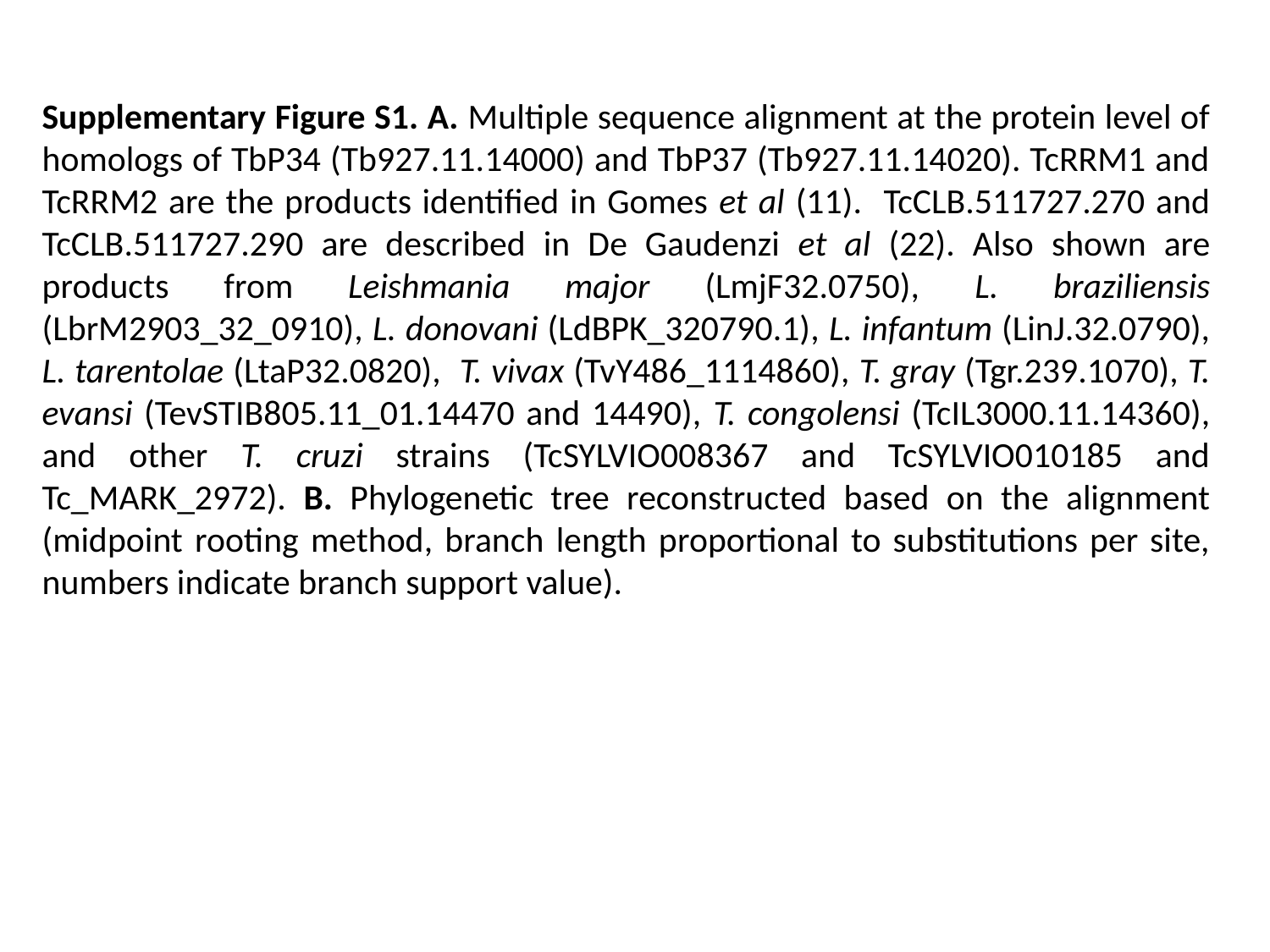

Supplementary Figure S1. A. Multiple sequence alignment at the protein level of homologs of TbP34 (Tb927.11.14000) and TbP37 (Tb927.11.14020). TcRRM1 and TcRRM2 are the products identified in Gomes et al (11). TcCLB.511727.270 and TcCLB.511727.290 are described in De Gaudenzi et al (22). Also shown are products from Leishmania major (LmjF32.0750), L. braziliensis (LbrM2903_32_0910), L. donovani (LdBPK_320790.1), L. infantum (LinJ.32.0790), L. tarentolae (LtaP32.0820), T. vivax (TvY486_1114860), T. gray (Tgr.239.1070), T. evansi (TevSTIB805.11_01.14470 and 14490), T. congolensi (TcIL3000.11.14360), and other T. cruzi strains (TcSYLVIO008367 and TcSYLVIO010185 and Tc_MARK_2972). B. Phylogenetic tree reconstructed based on the alignment (midpoint rooting method, branch length proportional to substitutions per site, numbers indicate branch support value).
